# Peculiarities of Low-Temperature Behavior of Liquids Confined in Nanostructured Silicon-Based Material

**DOI:** 10.3390/nano10112151

**Published:** 2020-10-28

**Authors:** Vladimir Bardushkin, Andrey Kochetygov, Yulia Shilyaeva, Olga Volovlikova, Alexey Dronov, Sergey Gavrilov

**Affiliations:** National Research University of Electronic Technology, Bld. 1, Shokin Square, Zelenograd, 124498 Moscow, Russia; bardushkin@mail.ru (V.B.); aakcht@gmail.com (A.K.); 5ilova87@gmail.com (O.V.); mfh.miet@gmail.com (A.D.); rnd@miee.ru (S.G.)

**Keywords:** electrode, electrolyte, nanostructured silicon, melting point depression, bulk density of strain energy, modeling, differential scanning calorimetry

## Abstract

This study is devoted to the confinement effects on freezing and melting in electrochemical systems containing nanomaterial electrodes and liquid electrolytes. The melting of nanoparticles formed upon freezing of liquids confined in pores of disordered nanostructured *n*-type silicon has been studied by low-temperature differential scanning calorimetry. Experimental results obtained for deionized water, an aqueous solution of potassium sulfate, and *n*-decane are presented. A model is proposed for predicting the melting point of nanoparticles formed during freezing of liquids inside the pores of a disordered nanostructured material. The model is based on the classical thermodynamic concept of the phase transition temperature dependence on the particle size. It takes into account the issues arising when a liquid is dispersed in a matrix of another material: the effect of mechanical stress resulted from the difference in the thermal linear expansion coefficients at a temperature gradient, the effect of the volumetric liquid content in the matrix, the presence of a nonfreezing liquid layer inside the pores, and the effect of wettability of the matrix with the liquid. Model calculations for water and *n*-decane confined in nanostructured silicon matrix have been carried out considering the volumetric liquid content. The results obtained have been compared with the differential scanning calorimetry data.

## 1. Introduction

Over the last decades, silicon-based anode materials for metal–ion batteries have been of great interest due to their tremendous theoretical specific capacity, accessibility of raw materials, economy, and environmental friendliness [1,2]. Thereby, lately, the researchers direct their efforts to correct the present deficiencies, such as relatively low electrochemical characteristics and significant volumetric changes that occur upon insertion and extraction of Li ions and could result in electrode destruction [3]. Several approaches are known today to resolve noted problems, for instance, using silicon in the form of thin films [4], spherical nanoparticles [5], one-dimensional nanostructures [6,7] and Si/C nanocomposites [8], as well as porous silicon, etc. [9]. It was noted that the use of nanostructured Si-based anode materials allows minimizing challenges posed by the volumetric changes and, at the same time, to increase the electrochemical characteristics [10,11]. One way or another, the resulting move towards nanoscale is accompanied by a significant increase in specific surface area. This may lead to various effects of nanoconfinement associated with the increased interface contact area in the electrode–electrolyte system. In the case of liquid electrolytes, such effects include, for example, remarkable changes in melting and freezing temperatures.

The properties of liquids under confinement have been extensively studied since the middle of the last century. At first, there were experimental investigations of supercooled water in the various systems, such as rocks [12], construction materials [13], and biological objects [14]. A large number of experimental studies have been associated with the phase transitions for a variety of different types of liquids inside disordered porous media, for instance, in zeolites [15], silica gels [16], activated carbons [17,18]. The vast majority of studies indicated the presence of a nonfreezing liquid layer bound to pore walls and changes in phase transition temperatures [19]. Due to nanotechnology’s rapid development, research of liquids under confinement has acquired greater importance. The phase transitions in confined water inside carbon nanotubes have already been studied [20]. Features of freezing/melting of water in the confined nanospace under an external stimulus were described in [21] based on data of differential scanning calorimetry (DSC). The DSC-method was also applied to investigate phase behavior of water inside pores of silica gel [22] and to examine some other liquids under nanoconfinement [23,24], as well as to determine pore size of different materials on the basis of Gibbs–Thomson equation [25,26,27]. This method, known as DSC-based thermoporosimetry, is applicable only with reference measurements with standard samples. Thus, the task of predicting the properties of confined liquids remains relevant.

It is also known that, in the case of a substantial temperature gradient, the confinement phenomena can be affected by the bulk density of strain energy [28,29]. This physico-chemical characteristic is caused by the mechanical stress resulting from the difference in the thermal linear expansion coefficients (TLECs) of contacting phases. In this paper, we proposed the model for predicting the melting point of nanoparticles formed during freezing of liquids inside the pores of a disordered nanostructured material accounting the bulk density of strain energy. Numerical model calculations of the melting point dependence on the pore size matrix have been carried out for water and *n*-decane confined in nanostructured silicon considering the volumetric liquid content. The results obtained are compared with the experimental DSC data. The influence of the measurement conditions and the matrix wettability with various liquids on the experimental results is discussed.

## 2. Experimental

### 2.1. Formation of Por-Si

Single-crystal *n*-type Si wafers with (111) orientation and a specific resistivity of 0.001 Ω·cm were used to form a nanostructured matrix. Nanostructured Si layers were prepared by electrochemical anodizing [30] in the galvanostatic mode for 120 min in an aqueous solution containing HF and glacial acetic acid with volume ratio 4:7. Pt was used as the cathode material. The current density was 15 mA·cm^−2^. To obtain porous silicon (por-Si) membranes, the nanostructured Si layers were detached from the substrate by incrementally increasing the current density by 10–15 mA·cm^−2^ every 3–5 min up to 62.2 mA·cm^−2^. Then the por-Si membranes were rinsed in ethanol and dried in air.

### 2.2. Characterization of Por-Si

Control of geometric parameters of por-Si membranes was performed using the scanning electron microscope Helios NanoLab 650 (FEI, Eindhoven, Netherlands) and surface area and pore size analyzer Quantachrome Nova 3200e (Quantachrome Instruments, Boynton Beach, FL, USA). Before the adsorption measurements, the samples were outgassed at 573 K for 3 h. Then N_2_ adsorption and desorption isotherms were measured at 77 K. All data analyses were performed using the NovaWin software (V.11.03 1994–2013, Quantachrome Instruments, Boynton Beach, FL, USA). The specific surface area was calculated in the relative pressure interval of 0.05–0.25 using the Brunauer–Emmett–Teller (BET) method. A cross-sectional area of 0.162 nm^2^ was used for the nitrogen molecule in the BET calculations. The pore size distribution was determined by DFT (Density Functional Theory) method.

The crystal structure of por-Si membranes was investigated by means of the “RIKOR-8” X-ray measuring system (“IRO”, Moscow, Russia). The measurements were done in an asymmetric geometry with CuK_α_ radiation (λ = 0.154 nm), a Ni filter was used for the K_β_-line stripping. The parallel beam focusing was used for 2θ scanning mode by a linear position-sensitive detector with an angular pitch Δ (2θ) = 0.02 deg, an incident angle θ = 6.00 deg. Scanning time was 500 s in a point.

The por-Si surface’s wettability by liquids studied was analyzed by the sessile-drop method using the goniometer LK-1 (OpenScience Ltd.) equipped with a digital camera. The 5.0× magnification was used. Measurements were carried out for deionized water (18.2 MΩ·cm), 0.6 M aqueous K_2_SO_4_ solution, and *n*-decane. The drop volume was 5 μL. Processing of images obtained applying goniometer was performed using the Drop Shape software.

### 2.3. Differential Scanning Calorimetry

Precalibrated differential scanning calorimeter Netzsch DSC 204 F1 Phoenix (Netzsch-Geratebau GmbH, Germany) equipped with mechanical cooling system Netzsch Intracooler was used to analyze the thermal behavior of liquids confined in the pores of por-Si membranes over the temperature range 193–293 K. The cooling and heating of the samples of 5–15 mg were carried out in hermetically sealed aluminum crucibles at a rate of 5 K/min. The sample mass was measured before and after DSC-analysis using an electronic balance Mettler Toledo XP 250 with an accuracy of 0.01 mg.

The saturation of porous media with liquids (deionized water, 0.6 M aqueous K_2_SO_4_ solution, and *n*-decane) was carried out immediately before DSC measurements. The por-Si fragments were wholly immersed in the studied liquids (deionized water, 0.6 M aqueous K_2_SO_4_ solution, and *n*-decane) and sonicated for about 3 min at 295 K until no more air bubbles escape. Also, the por-Si membrane was saturated with water vapor in a desiccator at room temperature for 24 h and analyzed by DSC under identical conditions.

## 3. Results

### 3.1. Structural Properties of the Nanostructured Si Matrices

**Figure 1:**
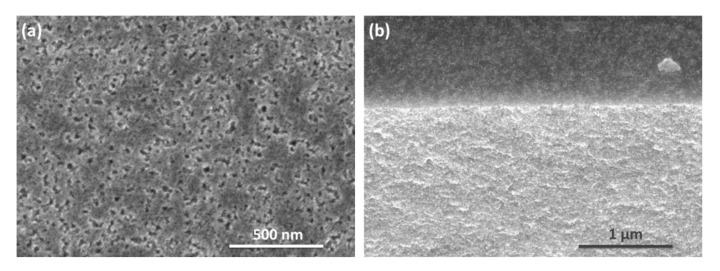
SEM top view (**a**) and a cross-sectional view and (**b**) of the por-Si membrane.

**Figure 2:**
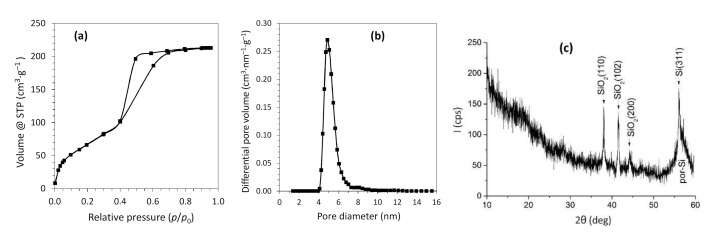
Isotherm of N_2_ adsorption-desorption at 77 K (**a**), pore size distribution (**b**), and X-ray diffractogram (**c**) of the por-Si membrane.

According to scanning electron microscopy images given in Figure 1, the electrochemical anodizing process results in the formation of disordered nanostructured porous Si layers. The thickness of por-Si membranes obtained is 80 ± 1 μm. Nitrogen adsorption/desorption analysis of membranes showed a capillary-condensation hysteresis on the isotherm (Figure 2a) which indicates the presence of end-to-end mesopores. The specific surface area calculated from experimental data was found to be 265.5 m^2^·g^−1^. The pore size distribution plot (Figure 2b) obtained by the DFT method reveals that the por-Si membranes are dominated by the pores with diameter of 4–8 nm.

The results of the XRD-analysis of the por-Si membranes are presented in Figure 2c. On the diffractogram, there are signals corresponded to the por-Si and SiO_2_ as a result of the porous silicon tendency to surface oxidation during storage in air. Nonlinear background in the range of 2Θ angles up to 30° is associated with X-rays scattering on the amorphous phase.

**Figure 3:**
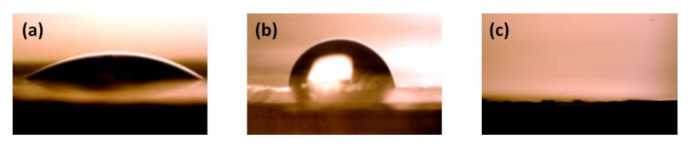
Sessile-drop measurements: pictures of droplets of (**a**) deionized water, (**b**) 0.6 M aqueous solution of K_2_SO_4_, and (**c**) *n*-decane on the surface of porous silicon membrane (5.0× magnification).

The results of wetting angle measurements for the por-Si membrane are given in Figure 3. It can be seen that the surface wettability by the water (Θ = 30° ± 3°) is higher than by the K_2_SO_4_ solution (Θ = 80° ± 3°), and, in the case of *n*-decane, there is a complete wetting of the surface (Θ = 0°). According to data obtained, por-Si membranes do not reveal pronounced hydrophobic properties, probably due to the surface SiO_2_ layer’s presence. This is in line with the available literature data on porous silicon wettability by various liquids [31]. In this regard, in the following, we will mainly consider the case of the complete wetting of the por-Si matrix by the liquid phase.

### 3.2. DSC Investigations

**Figure 4:**
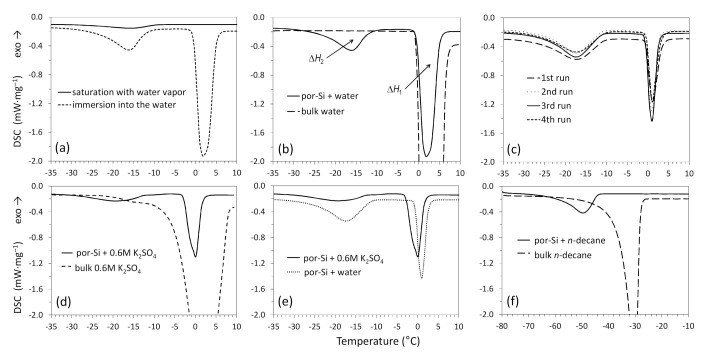
Differential scanning calorimetry (DSC) results: heating scans of the por-Si containing H_2_O (**a**–**c**); heating scan of the por-Si containing 0.6 M K_2_SO_4_ solution (**d**); comparison of scans of the por-Si membranes containing H_2_O and 0.6 M K_2_SO_4_ solution (**e**); and heating scan of the por-Si contained *n*-decane (**f**).

Low-temperature DSC was applied to analyze the melting of nanoparticles formed upon freezing of three different liquids confined in the por-Si membranes: deionized water, 0.6 M aqueous K_2_SO_4_ solution, and *n*-decane. Figure 4a–c reports the results of DSC measurements of por-Si membranes saturated with H_2_O after cooling down to 233 K.

Note that the scanning rate of 5 K·min^−1^ was selected based on the preliminary measurements as the minimum possible rate for detecting the thermal anomalies related to the melting of nanoparticles inside the matrix. The DSC signal shown by a dashed line in Figure 4a relates to the por-Si membrane saturated with water by immersion. It demonstrates two well-defined endothermic peaks caused by the melting of ice. The high-temperature peak corresponds to the melting of ice formed upon freezing of bulk water present in the crucible because of the sample preparation procedure. The second, low-temperature peak is caused by the melting of nanoparticles formed upon freezing of water confined in pores.

For comparison, the solid line in Figure 4a represents the DSC signal of por-Si membrane saturated with water vapor in a desiccator at room temperature for 24 h. In this case, the high-temperature peak caused by bulk ice melting is expectedly absent. The intensity of the peak caused by the melting of ice in pores is much lower due to less pore filling when preparing a sample in this way. Figure 4b shows the difference of DSC heating signals between the reference bulk water sample and the por-Si membrane immersed in water after previously cooling to 233 K. There is only one peak for the reference sample caused by the melting of bulk ice (dotted line). The temperature characteristics of observed DSC anomalies are given in Table 1.

The thermal behavior of water confined in a por-Si matrix during thermal cycling in the temperature range of 233–293 K was also studied. Figure 4c exemplifies the DSC heating segments combined into one plot, related to four successive heating/cooling cycles. A slight difference can be seen in the heat flux value between the first heating segment and others due to the thermal prehistory of the samples [32]. However, the peaks are reproduced accurately enough, and the temperature characteristics of peaks are not changed. Based on these results, the conclusion is that the por-Si contained H_2_O (volumetric content is 0.45) is stable during thermal cycling in the specified temperature range.

Figure 4d reports the DSC data for the por-Si membrane saturated with 0.6 M aqueous K_2_SO_4_ solution by immersion and the reference bulk sample of the same solution after previously cooling to 223 K. The solid line in Figure 4d relates to the por-Si contained K_2_SO_4_ solution. It can be seen that there are two endothermic peaks caused by the melting. The more intense and high-temperature peak corresponds to the melting of ice formed upon freezing of excess bulk liquid in the crucible. The phase transition inside the pores causes the second, more broad and less intense peak in the temperature range from −35 to −10 °C. The difference between the peak minimum temperatures was found to be 19 degrees. The DSC signal shown by a dashed line in Figure 4d relates to the heating of reference bulk the K_2_SO_4_ solution. There is one broad endothermic peak of complex shape caused by the melting. The pronounced shoulder on the left side of the peak can be attributed to the melting of near-eutectic compositions of the K_2_SO_4_–H_2_O system [33].

As is known, the freezing point of the solution is lower than the freezing point of the pure solvent. In this work, K_2_SO_4_ solution was chosen as an example of an aqueous electrolyte to assess the contribution of the cryoscopic effect to the melting point depression of nanoparticles formed upon freezing of liquids under confinement. Figure 4e shows the DSC data for por-Si membranes saturated with 0.6 M aqueous K_2_SO_4_ and deionized water. In the case of the solution, both observed peaks shift to the lower temperatures relative to the peaks of pure water. The melting temperature decrease **Δ*T*** for the K_2_SO_4_–H_2_O system confined in the pores is 1 degree higher than for water.

Figure 4f reports the DSC data for the por-Si membrane contained *n*-decane and the reference bulk *n*-decane after previously cooling to 193 K. The DSC signal shown by a dashed line relates to the heating of bulk *n*-decane. Only one endothermic peak is observed at temperatures close to the reference values caused by the melting of bulk *n*-decane. The solid line represents the DSC signal of the por-Si membrane contained *n*-decane. There is also only one endothermic peak shifted to the lower temperatures relative to the bulk *n*-decane. The temperature characteristics of peaks given in Table 1 show a decrease in melting point **Δ*T*** of 19 degrees for *n*-decane confined in the pores.

The comparison of melting point values measured by DSC and calculated using model proposed in this work (see Section 4) is given in Table 1. One can see that experimental values for H_2_O and *n*-decane are in a good agreement with respect to the predicted data.

## 4. Model

This section presents a model for predicting the melting temperature of nanoparticles formed during freezing of liquids confined inside the pores of a disordered nanostructured material. It considers the mechanical stresses arising due to the temperature gradient and the thickness of the nonfreezing liquid layer. Herewith, the developed model’s basic statements will be considered in detail using the por-Si–H_2_O system as an example.

According to the classical thermodynamical concepts, the melting point $T_{m}$ of the particle enclosed in a matrix of other material can be described by the following equation [34]:

(1) $$\frac{T_{m}-T_{m\;,\;\infty }}{T_{m\;,\;\infty }}=\frac{1}{\Delta H_{m\;,\;\infty }}\left(\frac{2(\gamma_{LW}-\gamma_{SW})}{r}\right)$$

where $T_{m\;,\;\infty }$ (K) is the melting point of the corresponding bulk material; $\Delta H_{m\;,\;\infty }$ (J·m^−3^) is the heat of fusion per unit volume; $\gamma_{LW}$ and $\gamma_{SW}$(J·m^−2^) are liquid/pore wall and solid/pore wall interface energies, respectively; and *r* (m) is the radius of the particle.

**Figure 5:**
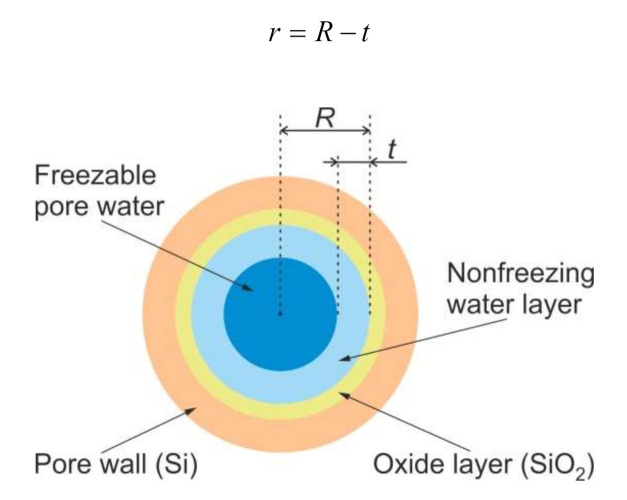
Model of water states in cylindrical mesopore of nanostructured Si.

Predicting the temperature of the solid–iquid phase transition under confinement in pores using Equation (1) requires accounting the presence of a nonfreezing liquid layer of thickness *t* [35,36]. Figure 5 shows the model of water states in the cylindrical mesopore of nanostructured Si. In this regard, the radius *r* of a particle freezing in a pore of a radius *R* can be represented as follows:

(2) r=R−t

In the case of complete wetting of the matrix pore walls by the liquid, the following assumption can be made [35]:

(3) $$\gamma_{LW}-\gamma_{SW}=\gamma_{LS}$$

where $\gamma_{LS}$ (J·m^−2^) is liquid–solid particle interface energy.

Taking into account the mechanical stresses resulted from the difference in the thermal linear expansion coefficients of matrix and particles at a temperature gradient [29,34] as well as Equations (2) and (3), it is possible to write Equation (1) in the following form:

(4) $$\frac{T_{m}-T_{m\;,\;\infty }}{T_{m\;,\;\infty }}=\frac{1}{\Delta H_{m\;,\;\infty }}\left(\Delta E+\frac{2\gamma_{LS}}{R-t}\right)$$

where ΔE (J·m^−3^) is the averaged value of bulk density of strain energy that is influenced by the composition and structure of matrix nanocomposite and the volumetric content of components [28,29,37].

The correct definition of ΔE requires one to introduce the notion of the operator of stress concentration $K^{\sigma }(r)$. Here r is the radius vector of a random point in the medium. This operator is a fourth-rank tensor [38] that connects the local values of stress tensor $\sigma_{ij}(r)$ with the nanocomposite average stresses $\langle \sigma_{kl}(r)\rangle$, i,j,k,l=1 , 2 , 3:

(5) $$\sigma_{ij}(r)=K_{ijkl}^{\sigma }(r)\langle \sigma_{kl}(r)\rangle$$

Angular brackets in Equation (5) and below define the statistical averaging procedure (over the volume or crystallographic axes orientations of crystallites) [38,39]. Herewith, for the multicomponent heterogeneous structures, averaging procedure over the volume for some random variable **b** is reduced to summing

(6) $$\langle b\rangle =\sum_{s}v_{s}\langle b_{s}\rangle$$

where $v_{s}$ is the volumetric content of the *s*-type component and $b_{s}$ is random variable corresponding to the specified component; $\sum_{s}v_{s}=1$ [38,39].

To determine $K^{\sigma }(r)$, there is a need to resolve the equilibrium equations for an elastic heterogeneous medium. However, in general, the ratio for numerical calculations is unfeasible to obtain. For this reason, the different approximations are applied for calculating $K^{\sigma }(r)$. One such approximation that considers the interaction of inclusions is the generalized singular approximation of the random fields theory [39]. In its context, only the singular component of Green’s tensor of the equilibrium equations is used that depends solely on the Dirac delta function. A homogeneous comparison body with material constants included in the final expressions for calculating $K^{\sigma }(r)$ is also introduced. The physical meaning of the generalized singular approximation is the assumption of homogeneity of the stress and strain fields within the element of heterogeneity. The expression for $K^{\sigma }(r)$ has then the following form (indices are omitted) [38]:

(7) $$K^{\sigma }(r)=c(r){\left(I-g(r)c^{\prime \prime }(r)\right)}^{-1}{\langle c(r){\left(I-g(r)c^{\prime \prime }(r)\right)}^{-1}\rangle }^{-1}$$

where **I** is the fourth-rank unit tensor and c(r) is the elasticity moduli tensor. The double primes indicate the difference between the parameters of a heterogeneous medium and a homogeneous comparison body, characteristics of which are denoted below by the superscript “*c*”:

$$c^{\prime \prime }(r)=c(r)-c^{c},$$

g(r) is the integral of the singular component of the second derivative of Green’s tensor of the equilibrium equations, which is a fourth-rank tensor. The components $a_{iklj}$ of the fourth-rank tensor **A** must first be calculated to determine the components of the tensor g(r). Then symmetrization procedure is performed using two pairs of indices (*i*, *j* and *k*, *l*) [39]. The components $a_{iklj}$ of tensor **A** can be calculated using the following expression:

(8) $$a_{iklj}=-\frac{1}{4\pi }\int n_{k}n_{j}\;t_{il}^{-1}d\Omega$$

where dΩ=sinθ dθdφ is an element of the solid angle in a spherical system of coordinates; $t_{il}^{-1}$ are the elements of the reverse matrix **T** with the elements $t_{il}=c_{iklj}^{c}n_{k}n_{j}$; and $n_{k}$ and $n_{j}$ (k , j=1 , 2 , 3) are the components of a vector of an external normal to the inclusion’s surface. For ellipsoidal inclusions with principal semiaxes $l_{1}$, $l_{2}$, and $l_{3}$, the components of the normal vector are determined by the relations:

$$n_{1}=\frac{1}{l_{1}}\sin\theta \cos\varphi ,\;n_{2}=\frac{1}{l_{2}}\sin\theta \sin\varphi ,\;n_{3}=\frac{1}{l_{3}}\cos\theta .$$

According to Equations (7) and (8), the operator $K^{\sigma }(r)$ depends only on the parameters of the material and structure of the heterogeneous medium.

The thermal expansion of matrix and inclusions was considered in this work as a factor leading to a change in the stress state of the entire system. In this case, the local stress values are [40]:

$$\sigma_{ij}(r)=c_{ijkl}(r)\alpha_{kl}(r)\Delta T,$$

where $\alpha_{kl}(r)$ are the thermal expansion tensor components and ΔT is the temperature change. For the heterogeneous structure considered in this work, the following expression can be written:

$$\alpha_{kl}(r)=\alpha (r)\delta_{kl},$$

where α(r) is the thermal expansion coefficient, with $\alpha (r)=\alpha_{H_{2}O}$ for inclusions and $\alpha (r)=\alpha_{\mathrm{Si}}$ for matrix; $\delta_{kl}$ is the Kronecker symbol.

The contribution of the local stress state of an individual inclusion to the average stress state of the nanocomposite is then [40]:

$$\langle \sigma_{s}\rangle ={\left(K_{s}^{\sigma }\right)}^{-1}\sigma_{s}={\left(K_{s}^{\sigma }\right)}^{-1}c_{s}\alpha_{s}\Delta T\;\delta_{kl}.$$

Hence, considering (6), the average stress caused by the thermal expansion of inhomogeneity elements will be determined by the following relation [40]:

(9) $$\langle \sigma \rangle =\left(\sum_{s}v_{s}{\left(K_{s}^{\sigma }\right)}^{-1}c_{s}\alpha_{s}\right)\Delta T\;\delta_{kl}$$

Equation (9) is applied for further model calculations of the energy characteristic ΔE, which is defined as the value resulting from the averaging of local bulk density of strain energy E(r) [28,29,37]:

(10) $$E(r)=\frac{1}{2}\varepsilon_{ij}(r)\sigma_{ij}(r)$$

In Equation (10), the composition of tensors of strain $\varepsilon_{ij}(r)$ and stress $\sigma_{ij}(r)$ is understood as the contraction on the corresponding indices.

To obtain Equation (10) in a convenient form for numerical calculations of ΔE, the generalized Hooke’s law is applied:

$$\varepsilon_{ij}(r)=s_{ijkl}(r)\sigma_{kl}(r),$$

where $s_{ijkl}(r)$ are the components of compliance tensor s(r). Then Equation (10) for E(r) can be written as:

(11) $$E(r)=\frac{1}{2}s_{ijkl}(r)\sigma_{kl}(r)\sigma_{ij}(r)$$

Bearing in mind Equation (5), we can rewrite Equation (11) as:

(12) $$E(r)=\frac{1}{2}s_{ijkl}(r)K_{klmn}^{\sigma }(r)\langle \sigma_{mn}(r)\rangle K_{ijpq}^{\sigma }(r)\langle \sigma_{pq}(r)\rangle$$

Thus, having designated $E(r)=E_{s}$ when calculating for the *s*-type heterogeneity element (oriented inclusion or matrix), we will obtain that, according to Equation (6):

(13) $$\Delta E=\sum_{s}v_{s}E_{s}$$

When modeling the nanocomposites structure in this work, the end-to-end mesopores were considered as strongly elongated ellipsoids of revolution equal to each other (with semiaxes $l_{1}$, $l_{2}$, and $l_{3}$). It was assumed that the ellipsoids are oriented by their major semiaxis in the space of the composite in five different directions relative to the laboratory rectangular coordinate system, namely: parallel to the vertical *z*-axis (one direction) and parallel to straight lines forming equal angles with all coordinate axes (four directions).

The inclusions of ice in the form of oriented filamentary particles were considered as a component of the first type. The silicon matrix was considered as a component of the second type. When performing numerical calculations, the nanocomposites were assumed to have the components with volumetric contents $v_{1}$ and $v_{2}$ ($v_{1}+v_{2}=1$), where the index “1” refers to inclusions, and “2”—to the matrix. In addition, it was assumed that inclusions have the same volumetric content $v_{1}/5$ in each of the five specified directions.

When carrying out numerical model calculations in operations with tensors, their matrix notation was used [39].

Monocrystalline Si used to form por-Si membranes is an anisotropic material with a cubic crystal structure. In this work, the following values were taken for the nonzero elements of the symmetric matrix **c** of the elastic moduli tensor of its single-crystals (GPa): $c_{11}=c_{22}=165.7$, $c_{12}=c_{13}=c_{23}=63.9$, and $c_{44}=c_{55}=c_{66}=79.6$ [39]. To carry out model calculations, we used the elastic characteristics of polycrystalline Si obtained by the self-consistency method [39]. These characteristics were determined by averaging over all possible orientations of the crystallographic axes of silicon crystallites (with an isotropic version of orientation distribution function), which reduces to integrating over all possible Euler angles [39]. For this purpose, an iterative procedure was organized in which the values of the elastic moduli tensor of polycrystalline Si obtained at the previous iteration step were taken as the comparison body’s parameters $c^{c}$. The elastic characteristics of polycrystalline Si obtained in the Hill approximation were taken as the initial values of the parameters of the comparison body [39]. The iterative procedure was terminated when the maximum difference between the modules $c^{c}$ was less than 0.01 GPa. It was assumed that the matrix $c^{c}$ obtained at the last step of the iterative procedure was the matrix of the elastic moduli tensor of polycrystalline Si, which was then used in calculations.

The same approach was applied to find the values of the matrix elements of the elastic moduli tensor of polycrystalline ice, which was used in further model calculations. In this case, for ice with a hexagonal crystal structure, the following values were taken for the nonzero elements of the symmetric matrix **c** of the elastic moduli tensor of its single crystals (GPa): $c_{11}=c_{22}=14.7$, $c_{33}=15.8$, $c_{12}=7.4$, $c_{13}=c_{23}=6.0$, $c_{44}=c_{55}=3.2$, and $c_{66}=0.5(c_{11}-c_{12})=3.65$ [41]. It should be noted that the values of the matrix elements of the elastic moduli tensor of polycrystalline ice calculated in this manner are in good agreement with the data presented in [42].

For the nanocomposites considered, according to Equation (6), Equation (7) for the operator of stress concentration in the *s*-type component has the following form [38]:

(14) $$K_{s}^{\sigma }=c_{s}{\left(I-g_{s}(c_{s}-c^{c})\right)}^{\;-1}{\left(\sum_{i}v_{i}c_{i}{\left(\;I-g_{i}(c_{i}-c^{c})\right)}^{\;-1}\right)}^{-1}$$

In Equation (14), $c_{s}$ and $c^{c}$ are the elastic moduli tensors of *s*-component of nanocomposite and the homogeneous comparison body, respectively; $g_{s}$ is the tensor g(r) of *s*-component of nanocomposite calculated by Equation (8). Here,$g_{2}$ corresponds to the silicon matrix $\left(l_{1}=l_{2}=l_{3}=1\right)$, $g_{1}$ corresponds to the oriented ellipsoidal ice inclusions (five different orientations; in model calculations, the value of the major semiaxis of the ellipsoids was taken equal to 4000, and the values of the others semiaxes were taken equal to 1).

When calculating the elastic characteristics $c^{c}$ of the homogeneous comparison body of por-Si–H_2_O nanocomposite, the self-consistency method was used [39]. An iterative procedure was applied in which the values of the elastic moduli tensor obtained at the previous iteration step were taken as the comparison body’s parameters $c^{c}$. The elastic characteristics obtained in the Hill approximation were taken as the initial values of the parameters of the comparison body [39]. The iterative procedure was terminated when the maximum difference between the modules $c^{c}$ was less than 0.01 GPa.

Then, for the considered por-Si–H_2_O nanocomposite, the bulk density of strain energy E(r) and the average density of strain energy ΔE were calculated depending on the volumetric content $v_{1}$ of oriented inclusions using Equations (12) and (13). To do this, the following TLECs values were taken (at T=243 K): $\alpha_{H_{2}O}=50.61\cdot 10^{-\;6}$$K^{-1}$ and $\alpha_{\mathrm{Si}}=1.99\cdot 10^{-\;6}$$K^{-1}$ obtained by cubic spline interpolation of data given in [43]. Calculations were carried out at T≥233 K and ΔT=20 K. When calculating E(r) by Equation (12), we used the fact that $s=c^{-1}$ [39] to find the values of elements $s_{ij}$ (i , j=1 , … , 6) of matrix **s** of compliance tensor.

**Figure 6:**
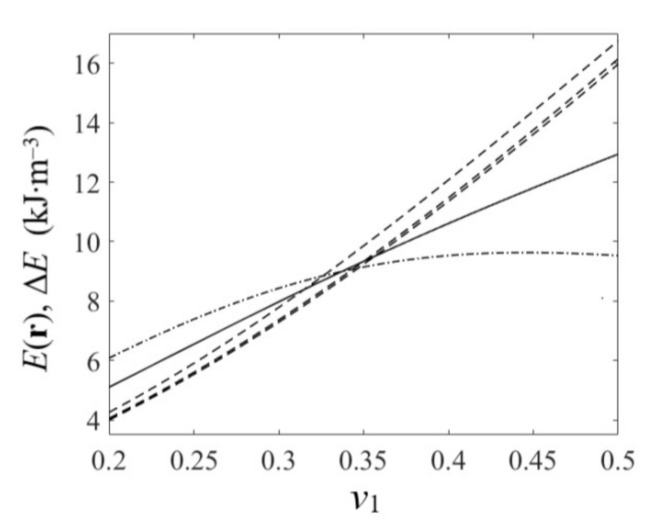
Bulk density of strain energy E(r) in H_2_O inclusions (dashed lines) and in the por-Si matrix (dash-dotted line), and average density of strain energy ΔE(solid line) vs. volumetric content $v_{1}$ of inclusions.

Figure 6 shows the estimated dependencies of the E(r) and ΔE on $v_{1}$ for the considered model nanocomposites. The dashed lines correspond to E(r) values in oriented inclusions of ice; the dash-dotted line corresponds to E(r) values in the por-Si matrix; and the solid line corresponds to the values of average density of strain energy ΔE.

**Figure 7:**
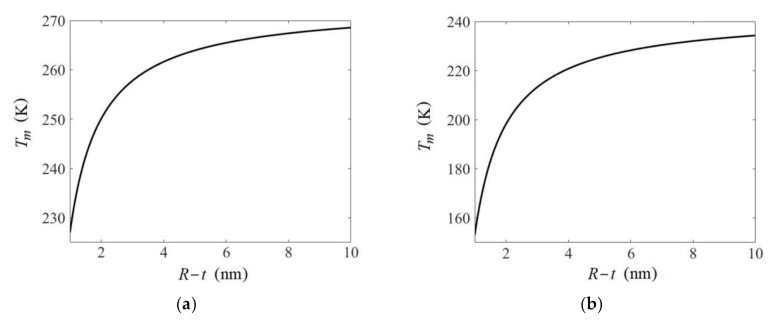
Melting temperature $T_{m}$ (K) vs. average radius r=R−t (nm) of nanoparticles of H_2_O (**a**) and *n*-decane (**b**) formed upon freezing of liquid confined in the por-Si.

The melting temperature $T_{m}$ of nanoparticles of H_2_O frozen in the por-Si matrix was calculated using Equation (4). Thermodynamic characteristics given in Table 2 and average density of strain energy ΔE=11.80 kJ·m^−3^ were used in model calculations for the volumetric content of H_2_O $v_{1}=0.45$ (See Figure 6). The results of numerical modeling of $T_{m}$ depending on average pore radius considering thickness *t* of the nonfreezing liquid layer are presented in Figure 7a.

Investigations that were conducted revealed that the contribution of mechanical stresses quantitatively expressed by the energy characteristic ΔE to the melting point change is negligible compared to surface contribution expressed by the ratio $\frac{2\gamma_{LS}}{R-t}$ in Equation (4). Similar findings were obtained in [29,37] when modeling the ΔE contribution in the melting point change of metallic nanoparticles in the anodic alumina matrix. In this regard, the modeling of $T_{m}$ depending on r=R−t for *n*-decane was performed neglecting the ΔE value. Thermodynamic characteristics given in Table 2 were used. The results obtained are given in Figure 7b.

The thickness *t* of the nonfreezing liquid layer was found using DSC data obtained for water applying the method proposed in [45]. Under this method, DSC allows to detect the thermal effects related only to the melting of freezable pore liquid and excess bulk liquid and to determine the corresponding phase transition heats. Therefore, knowing the total amount of liquid and true melting heat, one can calculate the contents of liquid fractions with different properties.

The total amount of water was determined by the difference of sample masses measured before and after the calorimetric experiment. For this purpose, the lid of the crucible was punched at the end of each DSC measurement to allow the evaporation of water when dried in an oven at 423–473 K. Thermal treatment was performed until achieving constant mass. The total water content $W_{t}$ was determined as the mass ratio of H_2_O to the dry por-Si. Then, the bulk water content $W_{b}$, freezable pore water content $W_{fp}$, and nonfreezable pore water content $W_{nf}$(expressed as the mass ratio of each content to the dry solid) were then calculated using the following equations [45]:

$$W_{b}=\frac{\Delta H_{1}\cdot m}{\Delta H_{0}},\;W_{fp}=\frac{\Delta H_{2}\cdot m}{\Delta H(T)},\;W_{nf}\;=W_{t}-W_{b}-W_{fp}.$$

Here, $\Delta H_{1}$ is the enthalpy change of fusion for bulk H_2_O and $\Delta H_{2}$ is the enthalpy change of fusion for freezable pore H_2_O, which are calculated by integrating the signal DSC in the temperature ranges of the corresponding endothermic peaks (Figure 4b). The true enthalpy change $\Delta H_{0}$ of bulk H_2_O at *T*_0_ is 334.1 J·g^−1^ [43]. The true enthalpy change ΔH(T)= 293.4 J·g^−1^ at 256 K (the temperature of a minimum of peak 2 in Figure 4b) was determined using the equation of Randall [35,45]. Calculations based on three independent DSC measurements of por-Si-H_2_O samples showed a reproducible ratio of $W_{fp}$ and $W_{nf}$ of 2.125:1. Therefore, considering that the membranes are dominated by the pores with a diameter of 4–8 nm, the thickness *t* of the nonfreezing H_2_O layer was found to be 0.4–0.7 nm. Considering the nonfreezing layer thickness independent on the liquid’s nature for a specific matrix material [36], were assumed the same values of *t* both for H_2_O and *n*-decane.

## 5. Conclusions

The model is presented that allows predicting the melting temperature of nanoparticles formed upon freezing of liquids confined in pores of disordered nanostructured material. Model calculations have shown that the contribution of mechanical stresses resulted from the difference in the thermal linear expansion coefficients at a temperature gradient to the melting point change is negligible compared to surface contribution. Low-temperature DSC data have shown the significant supercooling of liquids of different nature (water, water-salt system, and nonpolar organic liquid) confined in pores of nanostructured silicon. The melting point depression measured for H_2_O, aqueous K_2_SO_4_ solution, and *n*-decane was found to be 18, 19, and 19 K, respectively. The comparative analysis of experimental and calculated data showed satisfactory agreement of model calculations and measured values of the melting point. Low-temperature DSC analysis of the K_2_SO_4_–H_2_O system in the confined geometry conditions has also shown that the cryoscopic effect is smeared by the size effect of the melting point depression. Thus, the proposed model, combined with the presented DSC data, provides a useful framework for the discussion of the behavior of real electrochemical systems containing nanomaterial electrodes and liquid electrolytes at temperatures below 273 K.

## Figures and Tables

**Table 1:** DSC data on temperatures of extrapolated peak onset (***T_onset_***) and peak minimum (***T_peak_***) for melting of nanoparticles inside the pores in comparison with peak minimum temperature for melting of bulk substances.

Liquid	*T_onset_*, K	*T_peak_*, K	*T_peak,∞_*, K	Δ*T* *	*T_m,calc_*, K **
deionized water	247	256	274	18	247–257
0.6 M aqueous K_2_SO_4_ solution	242	254	273	19	–
*n*-decane	214	224	243	19	194–219

* The melting temperature decrease (**Δ*T***) is calculated as the difference between the peak minimum temperatures. ** The melting temperature values calculated using model proposed in this work (see Section 4) for the pores with diameter of 4–8 nm.

**Table 2:** Thermodynamic characteristics used in model calculations.

Liquid	$T_{m\;,\;\infty }\;(K)$ [43]	$\Delta H_{m\;,\;\infty }\;(\mathrm{kJ}\cdot mm^{-3})$ [43]	$\gamma_{LS}\;(J\cdot m^{-2})$
deionized water	273.15	306278	–0.0259 [36]
*n*-decane	243.30	153679	–0.0285 [44]
